# Di­chlorido­(4,7-dimeth­oxy-1,10-phenanthroline-κ^2^ *N*,*N*′)zinc(II)

**DOI:** 10.1107/S2414314624000373

**Published:** 2024-01-12

**Authors:** Nalani P. Rose, Hadi D. Arman, Rafael A. Adrian

**Affiliations:** a Department of Chemistry and Biochemistry, University of the Incarnate Word, San Antonio, Texas 78209, USA; b Department of Chemistry, The University of Texas at San Antonio, San Antonio, Texas 78249, USA; Vienna University of Technology, Austria

**Keywords:** crystal structure, zinc, 4,7-dimeth­oxy-1,10-phenanthroline, coordinating chloride ions, distorted tetra­hedral coordination environment, metal complex, τ_4_ descriptor

## Abstract

In the title complex, [ZnCl_2_(C_14_H_12_N_2_O_2_)], the Zn^II^ atom is located on a twofold rotation axis and is fourfold coordinated by two chlorido ligands and a bidentate 4,7-meth­oxy-1,10-phenanthroline ligand in a distorted tetra­hedral environment. Weak π–π stacking inter­actions between adjacent 4,7-dimeth­oxy-1,10-phenanthroline rings [centroid-to-centroid distances = 3.5969 (11) and 3.7738 (11) Å] contribute to the alignment of the complexes in layers parallel to (  01).

## Abstract 2

In the crystal structure of the title compound, the central zinc(II) metal atom is surrounded by two N atoms of a bidentate 4,7-meth­oxy-1,10-phenanthroline ligand and two chlorido ligands in a distorted tetra­hedral coordination environment.

## Graphical abstract

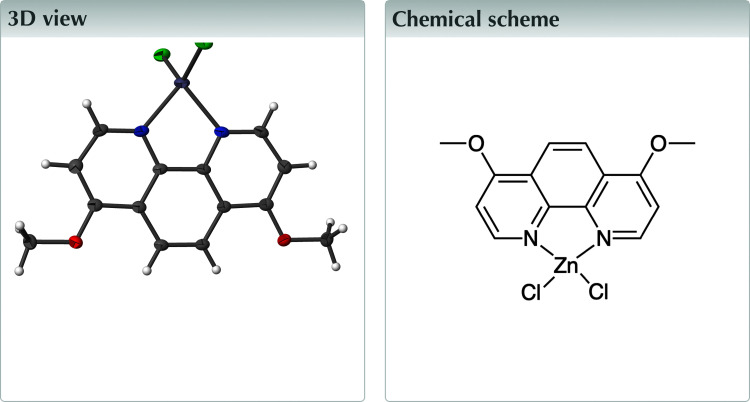

## Structure description

Over the last five years, metal complexes containing 4,7-dimeth­oxy-1,10-phenanthroline have garnered significant attention due to their catalytic activity (EL-Atawy *et al.*, 2018 ▸; Liu *et al.*, 2020 ▸) and potential as anti­tumor agents (Khoury *et al.*, 2022 ▸). Likewise, oxidovanadium(IV) complexes incorporating 4,7-dimeth­oxy-1,10-phenanthroline have been found to be effective against several cancer cell lines, including A2780 human ovarian adenocarcinoma and HCT116 human colorectal carcinoma (Choroba *et al.*, 2023 ▸). Currently, our research group focuses on creating metal complexes that have uses in biological systems. As part of this work, herein we present the synthesis and crystal structure of the title complex, which shows promise as a valuable precursor for the synthesis of novel zinc(II) complexes.

**Figure 1:**
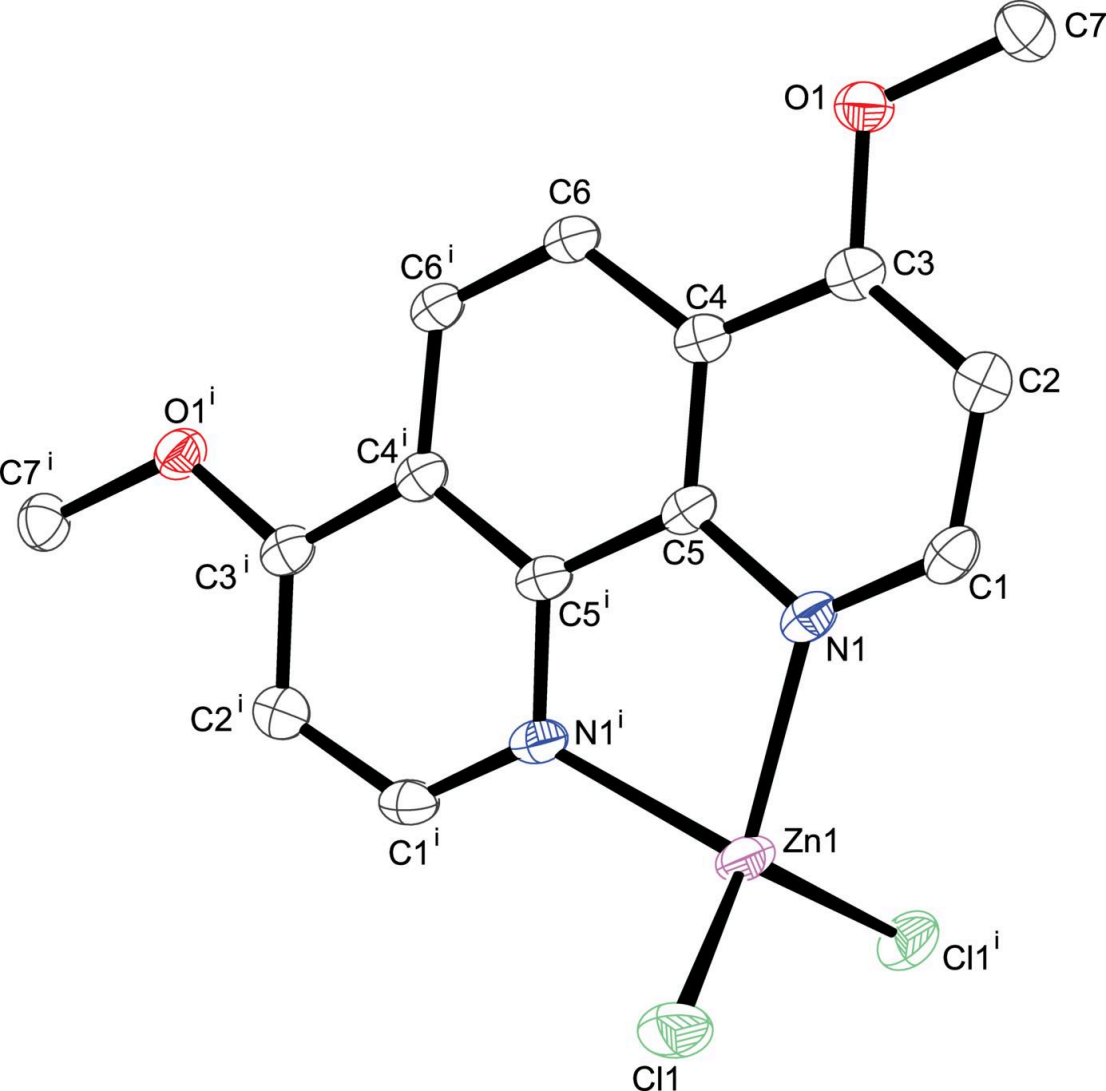
The mol­ecular structure of the title compound with displacement ellipsoids drawn at the 50% probability level; H atoms are omitted for clarity. Symmetry code: (i) −*x* + 1, *y*, −*z* + 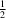 .

In the centrosymmetric crystal structure of the title complex, the zinc(II) atom is located on a twofold rotation axis (multiplicity 4, Wyckoff letter *e*) of space group *C*2/*c*. The coordination environment is that of a distorted tetra­hedron defined by two pyridine nitro­gen atoms from the 4,7-meth­oxy-1,10-phenanthroline ligand and two chlorido ligands (Fig. 1 ▸). The Zn—N bond lengths are in good agreement with comparable tetra­hedral 1,10-phenanthroline complexes currently available in the Cambridge Structure Database (CSD, version 5.45, Nov 2023; Groom *et al.*, 2016 ▸): refcodes DUCBOT (Niu *et al.*, 2009 ▸); TOBGOH (Li *et al.*, 2008 ▸); GODCOU (Luo *et al.*, 2019 ▸); QEVLIQ (Cetin *et al.*, 2020 ▸); ZNPHAT (Reimann *et al.*, 1966 ▸). At this time no 4,7-dimeth­oxy-1,10-phenanthroline zinc metal complexes have been deposited in the database. Similar behavior is observed for the Zn—Cl bond lengths. The τ_4_ descriptor value (Yang *et al.*, 2007 ▸) of 0.87 reflects the distortion from the perfect tetra­hedral coordination (τ_4_ = 1.0). Numerical data of relevant bond lengths and angles are presented in Table 1 ▸.

**Figure 2:**
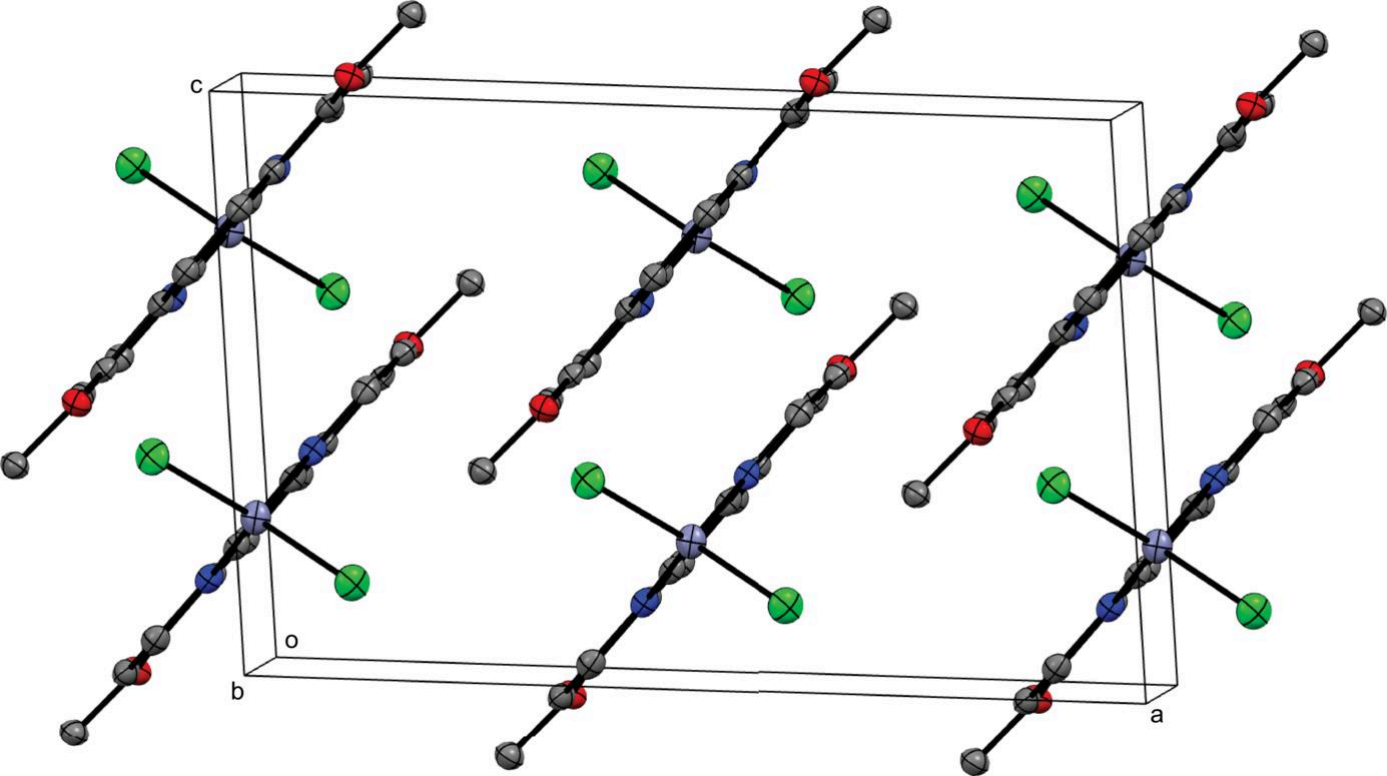
Perspective view of the crystal packing of the title complex approximately along the *b* axis; H atoms are omitted for clarity.

The title complex packs into layers parallel to ( 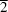 01) (Fig. 2 ▸). Contiguous pyridine rings show weak *π*–*π* stacking inter­actions, with centroid-to-centroid distances (*Cg*⋯*Cg*) alternating between 3.5969 (11) and 3.7738 (11) Å, and offset distances of 1.370 and 1.822 Å, respectively. No other significant supra­molecular inter­actions are present in the crystal packing of the title compound.

## Synthesis and crystallization

The title complex was synthesized by the addition of 4,7-dimeth­oxy-1,10-phenanthroline (0.176 g, 0.733 mmol) to a 40.0 ml aceto­nitrile suspension of zinc(II) chloride (0.100 g, 0.733 mmol). After the ligand was added, the resulting solution was heated at 333 K and stirred for 2 h. The resulting solution was then filtrated using a PTFE syringe filter to obtain a clear solution. Crystal suitable for X-ray diffraction were grown by vapor diffusion of diethyl ether over a satur­ated acetonitrile solution of the title complex.

## Refinement

Crystal data, data collection and structure refinement details are summarized in Table 2 ▸.

## Supplementary Material

Crystal structure: contains datablock(s) I. DOI: 10.1107/S2414314624000373/wm4203sup1.cif

Structure factors: contains datablock(s) I. DOI: 10.1107/S2414314624000373/wm4203Isup2.hkl

Supporting information file. DOI: 10.1107/S2414314624000373/wm4203Isup3.mol

CCDC reference: 2324427

Additional supporting information:  crystallographic information; 3D view; checkCIF report

## Figures and Tables

**Table 1:** Selected geometric parameters (Å, °)

Zn1—Cl1	2.2186 (5)	Zn1—N1^i^	2.0744 (18)
Zn1—Cl1^i^	2.2186 (5)	Zn1—N1	2.0744 (18)
			
Cl1—Zn1—Cl1^i^	120.75 (3)	N1^i^—Zn1—Cl1	107.88 (4)
N1^i^—Zn1—Cl1^i^	116.53 (4)	N1—Zn1—Cl1^i^	107.88 (4)
N1—Zn1—Cl1	116.53 (4)	N1^i^—Zn1—N1	80.66 (9)

Symmetry code: (i) 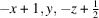 .

**Table 2:** Experimental details

Crystal data	
Chemical formula	[ZnCl_2_(C_14_H_12_N_2_O_2_)]
*M*_r_	376.53
Crystal system, space group	Monoclinic, *C*2/*c*
Temperature (K)	100
*a*, *b*, *c* (Å)	14.7877 (6), 9.9287 (4), 9.5230 (3)
β (°)	95.233 (4)
*V* (Å^3^)	1392.36 (9)
*Z*	4
Radiation type	Cu *K*α
μ (mm^−1^)	6.03
Crystal size (mm)	0.10 × 0.05 × 0.03

Data collection	
Diffractometer	XtaLAB Synergy, Dualflex, HyPix
Absorption correction	Gaussian (*CrysAlis PRO*; Rigaku OD, 2023 ▸)
*T*_min_, *T*_max_	0.780, 1.000
No. of measured, independent and observed [*I* > 2σ(*I*)] reflections	6451, 1385, 1282
*R*_int_	0.044
(sin θ/λ)_max_ (Å^−1^)	0.630

Refinement	
*R*[*F* ^2^ > 2σ(*F* ^2^)], *wR*(*F* ^2^), *S*	0.028, 0.078, 1.07
No. of reflections	1385
No. of parameters	97
H-atom treatment	H-atom parameters constrained
Δρ_max_, Δρ_min_ (e Å^−3^)	0.36, −0.54

Computer programs: *CrysAlis PRO* (Rigaku OD, 2023 ▸), *SHELXT* (Sheldrick, 2015*a* ▸), *SHELXL* (Sheldrick, 2015*b* ▸), and *OLEX2* (Dolomanov *et al.*, 2009 ▸).

## full crystallographic data

### Crystal data

**Table:**

[ZnCl_2_(C_14_H_12_N_2_O_2_)]	*F*(000) = 760
*M**_r_* = 376.53	*D*_x_ = 1.796 Mg m^−^^3^
Monoclinic, *C*2/*c*	Cu *K*α radiation, λ = 1.54184 Å
*a* = 14.7877 (6) Å	Cell parameters from 4164 reflections
*b* = 9.9287 (4) Å	θ = 4.6–76.0°
*c* = 9.5230 (3) Å	µ = 6.03 mm^−^^1^
β = 95.233 (4)°	*T* = 100 K
*V* = 1392.36 (9) Å^3^	Block, clear colourless
*Z* = 4	0.10 × 0.05 × 0.03 mm

### Data collection

**Table:**

XtaLAB Synergy, Dualflex, HyPix diffractometer	1385 independent reflections
Radiation source: micro-focus sealed X-ray tube, PhotonJet (Cu) X-ray Source	1282 reflections with *I* > 2σ(*I*)
Mirror monochromator	*R*_int_ = 0.044
Detector resolution: 10.0000 pixels mm^-1^	θ_max_ = 76.1°, θ_min_ = 5.4°
ω scans	*h* = −18→17
Absorption correction: gaussian (CrysAlisPro; Rigaku OD, 2023)	*k* = −10→12
*T*_min_ = 0.780, *T*_max_ = 1.000	*l* = −7→11
6451 measured reflections	

### Refinement

**Table:**

Refinement on *F*^2^	Primary atom site location: dual
Least-squares matrix: full	Hydrogen site location: inferred from neighbouring sites
*R*[*F*^2^ > 2σ(*F*^2^)] = 0.028	H-atom parameters constrained
*wR*(*F*^2^) = 0.078	*w* = 1/[σ^2^(*F*_o_^2^) + (0.0424*P*)^2^ + 1.5215*P*] where *P* = (*F*_o_^2^ + 2*F*_c_^2^)/3
*S* = 1.07	(Δ/σ)_max_ < 0.001
1385 reflections	Δρ_max_ = 0.36 e Å^−^^3^
97 parameters	Δρ_min_ = −0.54 e Å^−^^3^
0 restraints	

### Special details

**Table:**

Geometry. All esds (except the esd in the dihedral angle between two l.s. planes) are estimated using the full covariance matrix. The cell esds are taken into account individually in the estimation of esds in distances, angles and torsion angles; correlations between esds in cell parameters are only used when they are defined by crystal symmetry. An approximate (isotropic) treatment of cell esds is used for estimating esds involving l.s. planes.

### Fractional atomic coordinates and isotropic or equivalent isotropic displacement parameters (Å^2^)

**Table:**

	*x*	*y*	*z*	*U*_iso_*/*U*_eq_	
Zn1	0.500000	0.13609 (4)	0.250000	0.02301 (15)	
Cl1	0.39338 (4)	0.02563 (5)	0.35249 (5)	0.03050 (17)	
O1	0.66212 (10)	0.65250 (14)	0.53657 (14)	0.0218 (3)	
N1	0.56174 (11)	0.29536 (17)	0.36247 (16)	0.0199 (3)	
C5	0.53355 (13)	0.4178 (2)	0.30988 (19)	0.0182 (4)	
C4	0.56632 (13)	0.5410 (2)	0.36602 (19)	0.0183 (4)	
C6	0.53195 (13)	0.6646 (2)	0.30625 (19)	0.0189 (4)	
H6	0.553852	0.747773	0.345289	0.023*	
C2	0.66450 (14)	0.4095 (2)	0.5321 (2)	0.0218 (4)	
H2	0.711381	0.402937	0.606948	0.026*	
C3	0.63399 (13)	0.5341 (2)	0.48287 (19)	0.0194 (4)	
C1	0.62520 (13)	0.2938 (2)	0.4699 (2)	0.0214 (4)	
H1	0.645131	0.208835	0.506840	0.026*	
C7	0.73512 (14)	0.6509 (2)	0.6477 (2)	0.0239 (4)	
H7A	0.789459	0.611872	0.612157	0.036*	
H7B	0.717317	0.596632	0.726610	0.036*	
H7C	0.748220	0.743207	0.679964	0.036*	

### Atomic displacement parameters (Å^2^)

**Table:**

	*U*^11^	*U*^22^	*U*^33^	*U*^12^	*U*^13^	*U*^23^
Zn1	0.0248 (2)	0.0126 (2)	0.0319 (2)	0.000	0.00398 (15)	0.000
Cl1	0.0338 (3)	0.0212 (3)	0.0364 (3)	−0.0083 (2)	0.0030 (2)	0.0048 (2)
O1	0.0250 (7)	0.0181 (7)	0.0219 (7)	−0.0025 (6)	−0.0002 (5)	−0.0008 (5)
N1	0.0218 (7)	0.0143 (8)	0.0243 (8)	0.0015 (7)	0.0068 (6)	0.0027 (6)
C5	0.0202 (9)	0.0141 (10)	0.0211 (8)	0.0012 (8)	0.0075 (7)	0.0012 (7)
C4	0.0196 (8)	0.0168 (10)	0.0192 (8)	−0.0011 (7)	0.0063 (7)	−0.0004 (7)
C6	0.0208 (9)	0.0147 (9)	0.0220 (9)	−0.0008 (8)	0.0058 (7)	−0.0009 (7)
C2	0.0221 (9)	0.0235 (11)	0.0206 (8)	0.0013 (8)	0.0050 (7)	0.0021 (8)
C3	0.0213 (9)	0.0185 (10)	0.0196 (9)	−0.0016 (8)	0.0076 (7)	0.0013 (7)
C1	0.0234 (9)	0.0174 (10)	0.0241 (9)	0.0032 (8)	0.0059 (7)	0.0052 (8)
C7	0.0239 (9)	0.0263 (11)	0.0210 (9)	−0.0028 (9)	−0.0005 (7)	0.0004 (8)

### Geometric parameters (Å, º)

**Table:**

Zn1—Cl1	2.2186 (5)	C4—C3	1.429 (3)
Zn1—Cl1^i^	2.2186 (5)	C6—C6^i^	1.362 (4)
Zn1—N1^i^	2.0744 (18)	C6—H6	0.9500
Zn1—N1	2.0744 (18)	C2—H2	0.9500
O1—C3	1.334 (2)	C2—C3	1.383 (3)
O1—C7	1.441 (2)	C2—C1	1.395 (3)
N1—C5	1.365 (3)	C1—H1	0.9500
N1—C1	1.324 (3)	C7—H7A	0.9800
C5—C5^i^	1.442 (4)	C7—H7B	0.9800
C5—C4	1.404 (3)	C7—H7C	0.9800
C4—C6	1.427 (3)		
			
Cl1—Zn1—Cl1^i^	120.75 (3)	C6^i^—C6—C4	120.66 (11)
N1^i^—Zn1—Cl1^i^	116.53 (4)	C6^i^—C6—H6	119.7
N1—Zn1—Cl1	116.53 (4)	C3—C2—H2	120.6
N1^i^—Zn1—Cl1	107.88 (4)	C3—C2—C1	118.81 (19)
N1—Zn1—Cl1^i^	107.88 (4)	C1—C2—H2	120.6
N1^i^—Zn1—N1	80.66 (9)	O1—C3—C4	115.33 (18)
C3—O1—C7	117.29 (16)	O1—C3—C2	125.27 (18)
C5—N1—Zn1	112.59 (13)	C2—C3—C4	119.40 (19)
C1—N1—Zn1	129.60 (15)	N1—C1—C2	123.82 (19)
C1—N1—C5	117.74 (18)	N1—C1—H1	118.1
N1—C5—C5^i^	117.08 (11)	C2—C1—H1	118.1
N1—C5—C4	123.58 (18)	O1—C7—H7A	109.5
C4—C5—C5^i^	119.34 (11)	O1—C7—H7B	109.5
C5—C4—C6	119.99 (18)	O1—C7—H7C	109.5
C5—C4—C3	116.58 (18)	H7A—C7—H7B	109.5
C6—C4—C3	123.43 (18)	H7A—C7—H7C	109.5
C4—C6—H6	119.7	H7B—C7—H7C	109.5
			
Zn1—N1—C5—C5^i^	1.0 (2)	C6—C4—C3—O1	1.8 (2)
Zn1—N1—C5—C4	−178.96 (14)	C6—C4—C3—C2	−178.77 (17)
Zn1—N1—C1—C2	176.64 (13)	C3—C4—C6—C6^i^	179.3 (2)
N1—C5—C4—C6	−178.83 (16)	C3—C2—C1—N1	2.2 (3)
N1—C5—C4—C3	1.2 (3)	C1—N1—C5—C5^i^	178.14 (19)
C5—N1—C1—C2	0.1 (3)	C1—N1—C5—C4	−1.8 (3)
C5^i^—C5—C4—C6	1.2 (3)	C1—C2—C3—O1	176.58 (17)
C5^i^—C5—C4—C3	−178.81 (19)	C1—C2—C3—C4	−2.8 (3)
C5—C4—C6—C6^i^	−0.7 (3)	C7—O1—C3—C4	−175.50 (15)
C5—C4—C3—O1	−178.24 (15)	C7—O1—C3—C2	5.1 (3)
C5—C4—C3—C2	1.2 (2)		

Symmetry code: (i) −*x*+1, *y*, −*z*+1/2.
